# Role of thyroid hormones in burning mouth syndrome. Systematic review

**DOI:** 10.4317/medoral.25596

**Published:** 2022-09-29

**Authors:** Sonia Egido-Moreno, Joan Valls-Roca-Umbert, Mario Perez-Sayans, Andrés Blanco-Carrión, Enric Jane-Salas, José López-López

**Affiliations:** 1 Department of Odontoestomatology, Faculty of Medicine and Health Sciences, School of Dentistry, University Campus of Bellvitge, University of Barcelona, Barcelona, Spain; 2 Oral Medicine, Oral Surgery and Implantology Unit (MedOralRes), School of Medicine and Dentistry, University of Santiago de Compostela, Santiago de Compostela, Spain; 3 Oral Health and Masticatory System Group, IDIBELL (Bellvitge Biomedical Research Institute), University of Barcelona, Barcelona, Spain; 4 Faculty Director and Head of Service of the Medical-Surgical Area of Dentistry Hospital, University of Barcelona, Barcelona, Spain

## Abstract

**Background:**

Burning mouth syndrome is an idiopathic condition characterized by burning pain in a normal-appearing oral mucosa lasting at least four to six months. In the case of secondary burning mouth syndrome is associated with local or systemic factors (such as thyroid disorders) that can cause these symptoms. The aim of this review was to study the relationship between thyroid disorders and burning mouth syndrome.

**Material and Methods:**

The present study followed the PRISMA guidelines. An electronic search strategy was developed for PubMed/Medline, Scopus and Cochrane. The following combination of keywords and Boolean operators were used: Thyroid AND burning mouth; Thyroid AND burning mouth syndrome; Hypothyroidism AND burning mouth; Hypothyroidism AND burning mouth syndrome; Hyperthyroidism AND burning mouth; Hyperthyroidism AND burning mouth syndrome. The results were processed by existing free software in https://www.graphpad.com/. To evaluate the association of the categorical variables we used the Fisher test at a level of significance of *p-value* ≤ 0,05. As a primary summary measure the Odds Ratio (OR) has been used. To analyze the risk of bias the guidelines of the GRADE guide were used and the grade of evidence was analyzed by the guide of Joanna Briggs Institute: Levels of Evidence and Grades of Recommendations.

**Results:**

After applying the inclusion and exclusion criteria, 5 studies were selected for review. The Chi-square was 10.92 and the Odds Ratio was 3.31 with respect to TSH values with *p* <0.0001 (Fisher's test). The population of patients with TSH alterations is increased in 80.49% and decreased in 19.51%.

**Conclusions:**

It can be concluded that thyroid hormone abnormalities are a factor in secondary burning mouth syndrome; specially in patients with hypothyroidism.

** Key words:**Burning mouth syndrome, thyroid hormones, hypothyroidism.

## Introduction

Burning mouth syndrome is an idiopathic condition characterized by burning pain in a normal-appearing oral mucosa lasting at least four to six months (1). It is relatively common in middle-aged and elderly women, and it is estimated that the prevalence can reach 18% in postmenopausal women (2). BMS is characterized by burning sensations in the tongue (particularly at the tip and lateral edges), lips, palate (2), and gums (3). In addition to the burning sensation, it can also be accompanied by changes in taste and xerostomia (2-5). Symptoms must be present for at least 4 or 6 months continuously (2), but periods without pain during the day are also reported (4). In fact, many patients have no pain at night, but it progressively increases during the day (6).

To catalog the BMS, two classifications have been proposed. The first classification is based on symptoms fluctuations during the day. In Type 1, patients get up without pain and it increases throughout the day. It is related to systemic diseases, nutritional deficiencies or diabetes mellitus. In Type 2, patients have continuous symptoms during the day and have difficulty falling asleep. Usually associated with psychological disorders. In Type 3 the symptoms are intermittent and present periods of the day without pain (7).

The second classification is based on the etiology of the symptoms. Primary or essential BMS is idiopathic, there are alterations in the central/peripheral neuropathological pathways, but no causes are identified. The secondary is characterized by the presence of local or systemic alterations that can explain the symptoms (8). Systemic factors that can cause secondary BMS include deficiencies (iron, zinc, vitamin B12, folate) (3,5,7,9,10), hormonal disturbances (diabetes, thyroid disorders, anemia), use of certain medications (benzodiazepines, neuroleptics, antihypertensives) (5,9,10). For this reason, these factors must be excluded in the diagnosis of primary BMS (9,10). Regarding the etiology, local factors include alterations such as poor fit of the prosthesis, parafunctional habits, galvanism (3,7), allergic reactions, or xerostomia (3,5,7). Numerous studies have shown the association between BMS and psychological factors, such as depression and anxiety (4,6,7).

Regarding the pathogenesis of BMS, it is multifactorial, including neuropathic mechanisms at different levels of neuraxis that help explain the pathophysiology of BMS. Recent studies have shown that various peripheral neuropathic mechanisms contribute to the pathophysiology of primary BMS (2). In a study by Lauria *et al* (11) it was observed that patients with BMS presented a lower density of nerve fibers in the lateral border of the tongue than in patients without BMS, in addition to morphological changes are indicative of neuronal degeneration. Therefore, they concluded that BMS is a trigeminal small fiber sensory neuropathy. Other authors, such as Albuquerque *et al* (12) advocate the involvement of central neuropathic mechanisms. In their study, they demonstrate qualitative and quantitative alterations in central activation patterns. Using MRI, they observed less brain activity in patients with BMS.

The thyroid gland secretes triiodothyronine (T3) and thyroxine (T4) hormones that play an important role in tissue development and metabolism, as well as in the regulation of numerous functions and processes of the nervous system (13). Additionally, thyroid hormones have been shown to play a role in the maturation and specialization of taste buds (14). Therefore, the deficiency of these hormones can play a role in dysgeusia (15) and in peripheral/central neuropathies (13,15).

The objective of this review is to study the relationship between thyroid disorders and BMS.

## Material and Methods

The present study followed the Preferred Reporting Items for Systematic Reviews and Meta-Analysis (PRISMA) guidelines (16). A detailed protocol was prepared before starting the review and registered on Prospero (CRD42021248348)

- Hypothesis

Null Hypothesis (H0): Patients with thyroid disorders are not at increased risk of BMS

Alternative Hypothesis (H1): Patients with thyroid disorders are at increased risk of developing BMS

- Focused Question

We propose to review the existing literature asking the following question: Is the any association between thyroid alterations and the occurrence of burning mouth syndrome?

PECO Question: *P* (Population): Patients with BMS; E (Event): Thyroid disorders; C (Control): Patients without BMS; O (Outcome): Patients with thyroid disorders present higher prevalence of BMS.

- Eligibility Criteria

Inclusion criteria: case series, cross-sectional studies, cohort studies, case control studies, clinical trials, patients with thyroid disorders, human studies.

Exclusion criteria: Case studies, reviews; No restrictions were made for the year of publication, patients with previous thyroid pathology or other alterations/deficiencies; animal studies.

- Search strategy

An electronic search strategy was developed for PubMed/Medline, Scopus and Cochrane. A partial grey literature search was also performed. The following combination of keywords and Boolean operators were used: Thyroid [Mesh] AND burning mouth [tw]; Thyroid [Mesh] AND burning mouth syndrome [Mesh]; Hypothyroidism [Mesh] AND burning mouth [tw]; Hypothyroidism [Mesh] AND burning mouth syndrome [tw]; Hyperthyroidism AND burning mouth [tw]; Hyperthyroidism AND burning mouth syndrome [tw]. The lists of references of included studies were also hand-searched to identify additional relevant studies.

- Study selection

The last search was performed on March 5th, 2022. Two researchers (S.E.M and J.V.R) independently screened the title and abstract of every article identified in the search in order to establish its eligibility. A Coen’s kappa coefficient for each database was calculated to determine the reliability between researchers. Afterward, the full text of the selected articles was assessed for a definitive inclusion in the systematic review. A third reviewer (J.L.L.) resolved any discrepancies and agreed upon with S.E.M and J.V.R.

- Data Extraction y Statistical analysis

Data were extracted by the authors (S.E.M and J.V.R) and entered into a data collection form (Microsoft® Excel version 16.53) in which the data of Author(s), year of publication, number of patients, type of thyroid abnormalities (TSH, Anti-TPO, FT3, FT4, ultrasound abnormalities, TGA, GPCA, TMA).

These results were processed by the free software available at https://www.graphpad.com/. To evaluate the association of the categorical variables, we will use the Fisher test at a significance level of *p-value* ≤ 0.05. As a primary summary measure, the Odds Ratio (OR) will be used.

- Quality Assessment of Risk of Bias

To analyze the risk of bias, the guidelines of the guide “Grading of Recommendations, Assessment, Development and Evaluation” (GRADE) (17) and the Joanna Briggs Institute (JBI) Levels of Evidence and Grades of Recommendations, accessible at: https://jbi.global/sites/default/files/2019-05/JBI%20Levels%20of%20Evidence%20Supporting%20Documents-v2.pdf. [date 06-10-2022], referenced among other scientific works by that of Zhang *et al* (18), were used to analyze the degree of evidence.

## Results

- Results search strategy

**Figure 1:**
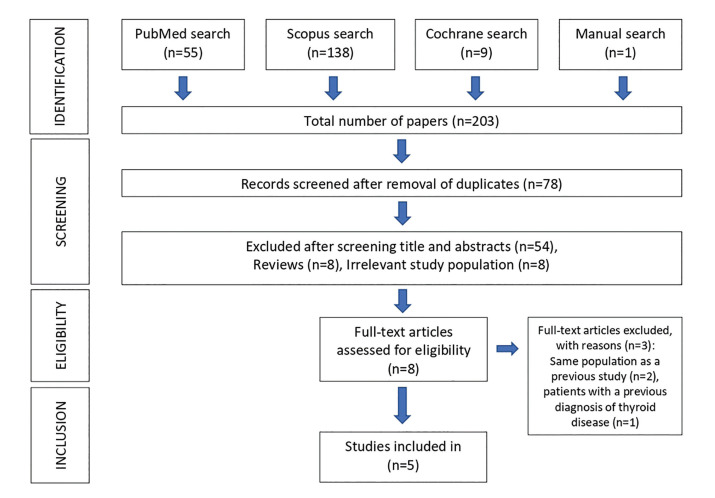
Flow diagram of literature search and selection criteria adapted from PRISMA.

A total of 203 articles were obtained using our search strategy. 125 articles were eliminated because they were repeated, of these 70 studies were excluded and 8 articles were selected for full text evaluation; 2 were excluded because the population was the same as that of another previous study and 1 study was excluded because the patients had a previous diagnosis of thyroid disease; so we finally selected 6 articles for the synthesis. The Cohen Kappa coefficient was 0.886 for the Pubmed search, 0.743 for Scopus, and 1 for the Cochrane search. Finally, 5 articles were chosen for the review plus 1 article that was chosen by manual search (3,15,19-21): 2 cross-sectional studies (3,15), 1 non-randomized clinical trial (20) and 2 case series (19,21) (Fig. 1).

- Study Results

The total population included 2,607 patients, of whom 1,992 had BMS versus 615 in the control group. Regarding gender, 3 studies inform us of the gender of the participants (15,19,21), the study by Femiano *et al* (20) does not inform us of the sex of the control group and the study by Cho *et al* (3) does not give us data regarding sex; finally, we found 1681 (76.13%) women versus 527 (23.87%) men. Making a distinction between the BMS group and the control group, in the BMS group we found 1,317 (76.75%) women versus 399 men (2.25%). In the control group, there are 364 (73.98%) women versus 128 (26.02%) men.

The mean age of the participants was 57.6±5.21, calculated in a population of 2,208 patients from 3 studies (15,19,21) plus the BMS group from the study by Femiano *et al* (20), the study by Cho *et al*. (3) did not report the age of the participants. Comparing the study group to the control group, in the BMS group (n=1666) the mean age of the participants was 62.98 years calculated in 3 studies (19-21) and that of the control group (n=442) was 57.5 years only reported by the study by Chiang *et al* (19) (Table 1).

**Table 1:**
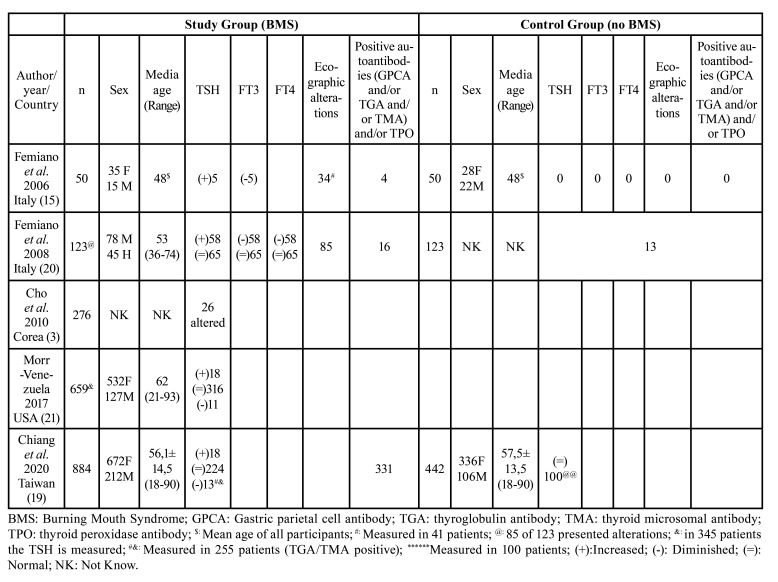
Study characteristics, analytic and ecographic alterations.

Thyroid abnormalities were evaluated by blood tests and ultrasound tests.

In the BMS group, the TSH hormone is evaluated by all the studies (3,15,19-21) (n=1049), although Chiang *et al* (19) only evaluated it in 255 patients. It is observed that 149 patients (14.2%) present alterations in this hormone. In the study by Cho *et al* (3) they only inform us that 26 patients present alterations in the hormone, but they do not specify whether there is an increase or decrease in it. In other studies (15,19-21), of a population of 123 patients with TSH abnormalities, 80.49% (n=99) had an increased level and 19.51% (n=24) had had a decreased level.

In the non-BMS group, the TSH hormone is evaluated by 3 studies (15,19,20), although the study by Chiang *et al* (19) only studied it in 100 patients out of 442; and the studies of (3,21) do not have a control group. The study population is 273 patients, of which 95.24% (n=260) do not have TSH abnormalities and 4.7% (n=13) have TSH abnormalities. Although they do not inform us of the type of alteration that there is.

The Chi-square was 10.92; therefore, the null hypothesis is rejected, and the Odds Ratio was calculated with respect to the TSH values; this was 3.31, so thyroid abnormalities are a risk factor for BMS; therefore, patients with thyroid abnormalities are 3.31 times more likely to have SBM than a patient without thyroid abnormalities and *p* <0.0001 (Fisher's test).

As for the other thyroid hormones, FT3 and FT4, ultrasound abnormalities or positivity for antithyroid antibodies, we do not have homogeneous data, so a quantitative analysis has not been possible. Although the article by Femiano *et al* (15) gave altered FT3, ultrasound and antibody values for the SBM group and found no alterations in the control group. The study by Feminano *et al* (20) found a higher proportion of alterations (FT3, FT4, ultrasound and antibodies) in patients in the SBM group than in those in the control group. And finally Chiang *et al* (19) focuses on the positivity of antithyroid antibodies, which in the SBM group are positive in 37.44% and in the control group they are positive in 17% (Table 1).

- Risk of bias

Studies were classified according to the GRADE and JBI system. According to the GRADE system; one study has a moderate methodological quality (20), two low (19,21) and the other two very low (3,15). Based on the JBI system, an article has a level 2.c. (20), two articles 4.b. (3,15) and the other two 4.c. (19,21).

## Discussion

This systematic review attempts to assess the relationship between thyroid abnormalities and burning mouth syndrome.

The literature estimates the prevalence of BMS between 0.7 and 5.1% (4), but in a review by Ariyawardana *et al* (22) on the definitions and criteria used in randomized clinical trials observed that only 19% of the trials used thyroid abnormalities as an exclusion criteria. Therefore, the data we have on primary/secondary BMS may be biased (22). The association of thyroid disorders and BMS is controversial. Since some authors are of the opinion that these alterations seem to have little influence on BMS and that the distinction between primary and secondary BMS is purely theoretical, a well as agreeing that BMS should be diagnosed only by the presence of symptoms excluding comorbidities. This theory is supported by the multimorbidity found in aging people, which increases in frequency due to the increase in life expectancy. It is alleged that the trials only focus on the target disease, excluding the rest of the pathologies, which makes it difficult to extrapolate the results to the real population (23).

Regarding age and gender, our review coincides with that published in the literature. In a recent review and meta-analysis on prevalence, a higher prevalence was found in women over 50 years of age (24).

The study by Femiano *et al* (20) proposes the term Burning mouth in hypothyroidism (BMHT) to differentiate them from cases of BMS, in which the burning sensation in the mouth corresponds to a symptomatic expression of hypothyroidism. In the current absence of evaluation of thyroid function and ultrasound, patients with true or subclinical hypothyroidism or only with ultrasound thyroid abnormality without thyroid abnormality are erroneously considered patients with BMS. Therefore, it is necessary to establish a protocol that includes thyroid function tests and ultrasound to differentiate patients with BMHT from real BMS (20).

The study by Talattof *et al* (25) studied the presence of BMS in patients with Hashimoto's thyroiditis. In which it was concluded that the levels of TSH, Anti-TPO, Anti-TG, Free T3 in these patients were related to the presence and severity of BMS. Treatment of this pathology can help prevent and treat these symptoms.

The symptoms of BMS in the literature relate them to hypothyroidism (20,25). This coincides with our results, in which it is observed that in 80% of the cases of TSH alteration, it is increased, which may indicate insufficient production of thyroid hormone, and therefore hypothyroidism.

Some patients, in addition to the sensation of burning, also present alterations in taste (26), this happens more frequently when there are thyroid alterations (20), in which, due to latent or subclinical hypothyroidism, they are more predisposed to dysgeusia or ghost tastes (15). Thyroid hormones are related to the maturation and specialization of the taste buds (14). Braud *et al* (27) in their study showed altered taste sensitivity in BMS patients, with increased thresholds of fungiform and foliate taste buds. The taste buds are surrounded by a collection of pain neurons from the trigeminal nerve (6). With reduced taste, loss of inhibition of pain afferent fibers occurs. Therefore, the origin of dysgeusia is due to the imbalance between somatosensory and gustatory afferent stimuli (28). In addition to an increased sensitivity of the trigeminal nerves (25).

The main limitation of our review was the heterogeneity of the data from the included studies, which did not allow us to perform a meta-analysis. In addition, some of the included studies did not have a control group (reference) and generally have poor methodological quality, which can lead to biased results.

## Conclusions

After carrying out this systematic review, we can conclude that alterations in thyroid hormones are a factor in secondary burning mouth syndrome; especially in patients with hypothyroidism. Although it should be corroborated with studies of higher methodological quality in which all the analytical aspects in relation to thyroid alterations were evaluated.
